# miR-15/107 microRNA Gene Group: Characteristics and Functional Implications in Cancer

**DOI:** 10.3389/fcell.2020.00427

**Published:** 2020-06-17

**Authors:** Chiara Turco, Sara Donzelli, Giulia Fontemaggi

**Affiliations:** Oncogenomic and Epigenetic Unit, Department of Diagnostic Research and Technological Innovation, IRCCS Regina Elena National Cancer Institute, Rome, Italy

**Keywords:** miR-15/107, miR-195, miR-497, miR-15, miR-107, miR-16, miR-503, Granulin (GRN)

## Abstract

The miR-15/107 group of microRNAs (miRNAs) encloses 10 annotated human members and is defined based on the presence of the sequence AGCAGC near the mature miRNAs’ 5′ end. Members of the miR-15/107 group expressed in humans are highly evolutionarily conserved, and seven of these miRNAs are widespread in vertebrate species. Contrary to the majority of miRNAs, which recognize complementary sequences on the 3′UTR region, some members of the miR-15/107 group are peculiarly characterized by the ability to target the coding sequence (CDS) of their target mRNAs, inhibiting translation without strongly affecting their mRNA levels. There is compelling evidence that different members of the miR-15/107 group regulate overlapping lists of mRNA targets but also show target specificity. The ubiquitously expressed miR-15/107 gene group controls several human cellular pathways, such as proliferation, angiogenesis, and lipid metabolism, and might be altered in various diseases, such as neurodegenerative diseases and cancer. Intriguingly, despite sharing the same seed sequence, different members of this family of miRNAs may behave as oncomiRs or as tumor suppressor miRNAs in the context of cancer cells. This review discusses the regulation and functional contribution of the miR-15/107 group to the control of gene expression. Moreover, we particularly focus on the contribution of specific miR-15/107 group members as tumor suppressors in breast cancer, reviewing literature reporting their ability to function as major controllers of a variety of cell pathways and to act as powerful biomarkers in this disease.

## Introduction

**FIGURE 1:**
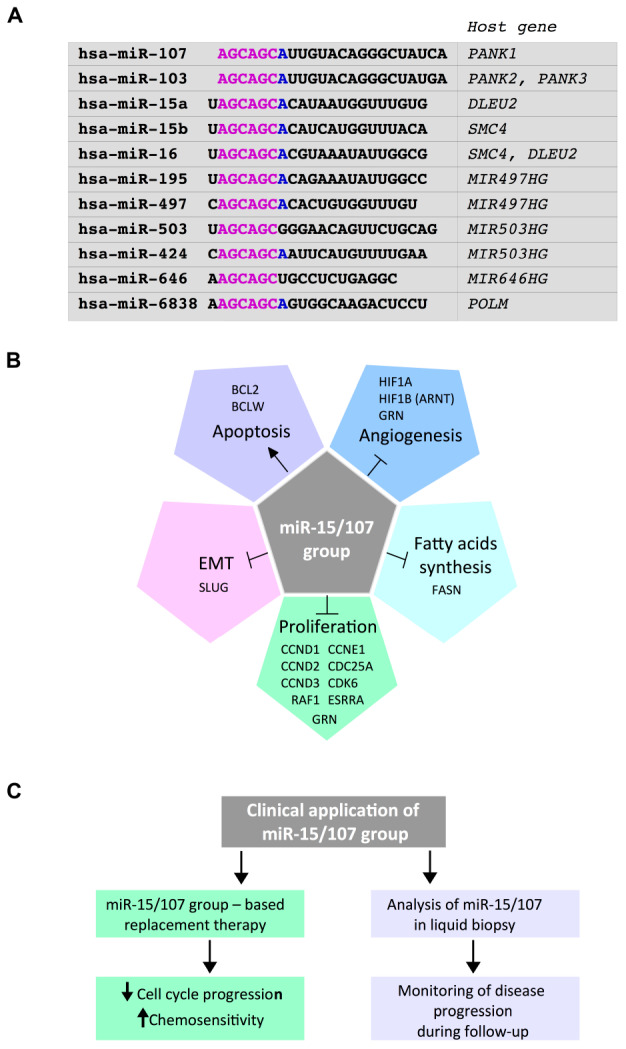
**(A)** Members of the miR-15/107 gene group are highly conserved and share an AGCAGC hexamer in their “seed” region. All members of the miR-15/107 group are enclosed in host genes, indicated on the right. **(B)** Diagram describing the main tumor suppressive functions exerted by members of the miR-15/107 group in breast cancer. Experimentally demonstrated targets relevant to the various functions are indicated. **(C)** Diagram describing putative fields of application of the miR-15/107 group members in the clinical practice in breast cancer. Delivery of modified oligonucleotides mimicking members of this microRNA (miRNA) group could strongly impair the proliferative potential of breast cancer cells and increase the response to therapies. In addition, evaluation of the levels of various miR-15/107 group members in liquid biopsy is a promising approach for the diagnosis and the monitoring of disease progression, especially for triple-negative breast cancer.

The miR-15/107 gene group contains multiple highly conserved microRNA members, including miR-15a-5p, miR-15b-5p, miR-16-5p, miR-103a-3p, miR-107, miR-195-5p, miR-424-5p, miR-497-5p, miR-503-5p, and miR-646 (Finnerty et al., 2010). The nomenclature referred to these molecules is not uniform in the literature, and six of these miRNAs (miR-15a/b, miR-16, miR-195, miR-424, and miR-497) are frequently referred to as miR-16 family members (Rissland et al., 2011). Inclusion in the miR-15/107 group is based on the presence of “AGCAGC” in the “seed” region starting at either the first nucleotide or the second nucleotide from the 5′ end of the mature (∼22 nt, single stranded) miRNA (Figure 1A). Recently, it has been evidenced that also miR-6838-5p contains the AGCAGC sequence in its seed sequence, and this miRNA has then been included as a new member of the miR-15/107 group (Wang F. et al., 2019). miR-15/107 family members are only expressed in chordates, with several being mammal specific (miR-195,-497, -503, -424, and -646) and miR-646 appearing only in humans and chimpanzees (Finnerty et al., 2010; Kozomara and Griffiths-Jones, 2014; Wang S. et al., 2019).

All members of this group are interestingly located in host genes (Figure 1A), including: (a) coding genes, as *PANK1* (containing miR-107), *PANK2* and *PANK3* (containing miR-103a-3p), *SMC4* (containing miR-15b-5p and miR-16-5p), *DLEU2* (containing hsa-miR-15a-5pand miR-16-5p), *POLM* (containing miR-6838-5p); (b) non-coding genes, as *MIR497HG* (containing miR-497-5p and miR-195-5p), *MIR503HG* (containing miR-503-5p and miR-424-5p and contained in the H19X locus), and *MIR646HG* (containing miR-646) (Finnerty et al., 2010; Necsulea et al., 2014). The conserved genomic organization of this group in four pairs of neighboring miRNA clusters, including miR-15a-5p/miR-16-5p, miR-15b-5p/miR-16-5p, miR-195-5p/miR-497-5p, and miR-424-5p/miR-503-5p within the common host genes, suggests that the miR-15/107 group members might be transcribed in pairs, except for the miR-646 and miR-6838-5p. Moreover, as both miR-103a-3p and miR-107 are hosted into the pantothenate kinase family (PANK) genes, even though located at entirely different chromosomes, they might be possibly subjected to common regulatory mechanisms. It has been reported that intronic miRNAs tend to be coexpressed with their protein-coding “mother” genes, although this is not always the case (Baskerville and Bartel, 2005; Monteys et al., 2010). Supporting a common phylogenetic origin, miR-15/107 family members have similar expression patterns and functions. The 5′ end sequence homology confers similar specificity in terms of targeting mRNAs for posttranscriptional decay and/or translational inhibition (Finnerty et al., 2010). miRNAs from this group are expressed in a wide variety of tissues, and given that many of their validated targets are involved in cell cycle, metabolism, and angiogenesis, it follows that dysregulation of these miRNAs is a hallmark of many disease states (Aqeilan et al., 2010; Li et al., 2011; Furuta et al., 2013).

## miR-15/107 Group Expression Patterns and Target Specificity

While all vertebrates examined to date express miR-15a, miR-15b, miR-16, miR-103, and miR-107, only mammals are known to express miR-195, miR-424, miR-497, miR-503, whereas miR-646 appears to be human specific (Finnerty et al., 2010). Overall, it can be considered that the miR-15/107 gene group of miRNAs is ubiquitous in that, as far as we know, no human cell type lacking the expression of at least one miR-15/107 gene group member has been described. Expression of mature miRNA forms of miR-15/107 family has been recently analyzed on 11 human tissues. Overall, these miRNAs were found highly expressed in a variety of these tissues including the brain, heart, lung, liver, kidney, spleen, stomach, and skeletal muscle (Wang et al., 2014). From RNA isolated from human brain samples, miR-16, miR-103, miR-107, and miR-497 are the most highly expressed miRNAs among the family members. Moreover, analysis of human tissues interestingly showed that the miRNAs apparently expressed at the highest levels include the 7-nt common sequence AGCAGCA (Finnerty et al., 2010; Wang et al., 2014). Analysis of tissue samples evidenced that individual members of the miR-15/107 group are expressed at medium-to-high levels across many tissue types, but there is some tropism in terms of both tissue- and cell type-specific expression. However, these data reveal that there is not, among these miRNAs, a sharply tissue- or cell-specific miR-15/107 gene. Interestingly, analysis of miRNA precursors in the same tissues showed that correlations between the expression of mature miRNA and that of the precursor transcript (pri-miRNA) were generally not strong for most miRNAs of the miR-15/107 group.

With regard to the ability of miRNAs belonging to this group to target mRNAs, a number of studies focused on the dissection of their shared functions, attributable to the presence of the common AGCAGC hexamer in the seed sequences, as well as of their specific functions, conferred by the rest of each miRNA sequence besides the AGCAGC. The group of Peter T. Nelson (University of Kentucky) contributed extensively to this topic leading to the publication of seminal papers on the characterization of the miR-15/107 group function. In the attempt to better understand the implications of both 5′ and 3′ portions of miRNA in terms of mRNA targeting, Nelson and colleagues considered the target mRNAs that are associated with AGO protein in RIP-Chip experiments, and also those mRNAs downregulated following transfections of miR-103, miR-107, miR-16, and miR-195 in cultured H4 glioneuronal cancer cells (Nelson et al., 2011). Analysis of recruited mRNAs showed that, as expected, miRNA 5′ seeds appear to be a critical targeting determinant; however, in contrast to the majority of previously reported miRNAs that predominantly target the 3′-UTR, miR-103 and miR-107 preferentially bind to the CDS (coding sequence) of the target transcript. This property depends on the 3′ end of the miRNA, which pairs with target mRNA. Indeed, mutation of the 3′ portion of the miRNA impairs its preference for CDS vs. 3′-UTR. Interestingly, authors identified, in the 3′ portion of miR-107, a sequence motif AGCCCUGU that was significantly enriched in a group of 110 genes targeted by miR-107. Within this motif, three adjacent pentamer words were significantly enriched, with anti-sense sequences found in 33 of the 110 target genes.

Concerning the identification of common regulatory programs driven by different members of this miRNA group, the comparison of transcriptome modulations caused by various members of miR-15/107 group with relative AGO-interacting mRNAs, indicated that miR-16, miR-103, miR-107, and miR-497 share high degrees of overlap in targets, with 20% of mRNA targets shared by all four miRNAs in H4 glioneuronal cell line (Nelson et al., 2011). On the same line, Wang and colleagues recently analyzed putative and validated target mRNAs of miR-15/107 group to dissect collective and specific functions of these miRNAs through bioinformatic and experimental approaches (Wang S. et al., 2019). In agreement with results previously obtained by Nelson’s group, their study showed the existence of a massive overlapping of target mRNAs among the miR-15/107 family; indeed, dozens of target genes could be affected by this family collectively, being subjected to regulation from more than 5 members of this family. Among the target mRNAs of the miRNA group, the most significantly regulated pathways included fatty acid metabolism/biosynthesis/degradation/elongation,various signaling during carcinogenesis, and some crucial pathways for cell survival such as cell cycle. The same study highlighted that, considering the validated targets, there are three pairs of miRNAs, including miR-15a-5p/miR-15b-5p, miR-103a-3p/miR-107, and miR-424/miR-497, closely resembling each other, while three of them, including miR-6838-5p, miR-503-5p, and miR-646, vary independently.

## Functional Relevance of miR-15/107 Group in Cancer

### Tumor Suppressor Activity of the miR-15/107 Group and the Control of the Cell Cycle

The deletion or downregulation of both miR-15a and miR-16 in cases of B cell chronic lymphocytic leukemias (B-CLL) was the first evidence that suggested that these members of the miR-15/107 group could act as tumor suppressors (Calin et al., 2005; Cimmino et al., 2005). The formal demonstration came from *in vivo* experiments showing that, in mouse models, deletion of the genomic 13q14 region, which encodes the miR-15a/16-1 locus, recapitulates B-CLL phenotypes observed in humans (Klein et al., 2010). Starting from those initial observations, numerous additional evidences concerning the downregulation of members of this group of microRNAs in cancer reinforced the hypothesis of their role as tumor suppressors. Indeed, they have been found often downregulated in a variety of cancer types, such as colorectal cancer, prostate cancer, mantle cell, and other non-Hodgkin B-cell lymphomas, lung adenomacarcinomas, and breast cancer (Calin et al., 2005; Bonci et al., 2008; Bandi et al., 2009; Klein et al., 2010; Liu et al., 2010; Zhang et al., 2012; Bertoli et al., 2015). Interestingly, miR-15a/miR-16-1 downregulation was shown to rely on the repressive activity of transcription factor Myc in B-cell lymphomas (Zhang et al., 2012).

Restoration of the expression of some of these downregulated miRNAs in cancer cells represses the tumorigenicity of cells, impinging on cell cycle and apoptosis (Liu et al., 2010). However, important differences in these effects were observed depending on the cell types analyzed. For example, Cimmino et al. showed that miR-15a and miR-16-1 induce apoptosis through the downregulation of the antiapoptotic gene BCL2 in leukemic cells (Cimmino et al., 2005). On the contrary, miR-16 expression caused G0/G1 accumulation without evidence of apoptosis in colon, lung, breast, and ovarian cancer cells (Linsley et al., 2007). A huge number of reports evidenced the strong impact of miR-15/107 group on cell cycle regulatory genes, especially genes controlling the G1/S transition, as Cyclin D1, D2, D3, Cyclin E1, CDC25A, and CDK6 (Cimmino et al., 2005; Linsley et al., 2007; Bonci et al., 2008; Liu et al., 2008; Marasa et al., 2009; Figure 1B).

In addition to the control exerted by these miRNAs on cell cycle progression, it has been reported that the expression of this miRNA group is, in turn, regulated in response to cell cycle changes (Rissland et al., 2011). Specifically, miR-15a, -15b, -16, -424, and -503 are dynamically upregulated during serum starvation and contact inhibition, with miR-503 showing the highest fold change, thus reinforcing the cell cycle arrest through the targeting of cell cycle-promoting genes, such as cyclins and CDKs. Conversely, as cells are released from G0 arrest, levels of some miR-16 family members rapidly decrease. Interestingly, while miRNAs are generally considered quite stable molecules, authors here demonstrated the intrinsic instability of some members of the miR-15/107 group, with miR-503 showing the highest instability. Specifically, the seed region and 3′ end of miR-503 were coordinately required for its instability. Identification of the nucleases responsible for miR-503 degradation remains an important question. The possible contribution of nucleases involved in cell cycle control, as for example the members of MCPIP family of endonucleases (Miekus et al., 2019), to the downregulation of miR-15/107 group during serum starvation is certainly a fascinating field that merits to be explored.

According to the tumor suppressor activity that might be exerted by these miRNAs, various studies highlighted that miR-107 expression may be induced by p53. Initially, miR-107 has been identified as a transcriptional target of p53 by Yamakuchi et al. (2010), who showed in colon cancer cells that p53, by inducing miR-107 expression, negatively impacts on angiogenesis, thanks to the ability of miR-107 to target HIF-1beta (also known as ARNT). Subsequently, miR-107 has been found upregulated by p53 also in glioma cells, where this miRNA is responsible for the inhibition of proliferation through the targeting of CDK6 and Notch-2 (Chen et al., 2013). Moreover, it has been shown that lethal doses of stress induce p53-dependent upregulation of both miR-103 and miR-107, which, in turn, inhibit LRP1 translation thus promoting cancer cell death (Leslie et al., 2018). Interestingly, a very recent report shows that, in the liver, p53 is responsible for the transcriptional induction of miR-107 and of its host gene *PANK1*, which contribute, respectively, to high−fat diet-induced insulin resistance and metabolic reprogramming (Yang et al., 2020).

Contrary to the positive transcriptional regulation of miR-107, a growing number of reports are currently showing that miR-107 activity is tightly controlled by sponging molecules, such as, for example, circular RNAs, that inhibit miR-107 tumor suppressor activity in cancer cells. Specifically, two circRNAs, namely, cTFRC and circTCF25, were reported to sequester miR-107 in bladder cancer cells, thus favoring cell cycle progression (Zhong et al., 2016; Su et al., 2019), while in gastric cancer, circHIPK3 was shown to sponge miR-107, enabling the release of brain-derived neurotrophic factor (BDNF) expression (Wei et al., 2020).

### miR-15/107 Expression and Function Are Altered in Breast Cancer

The miR-15/107 group members have been also reported to be modulated and functionally relevant in breast cancer (BC). Various studies and a meta-analysis recently reported miR-195 and miR-497 as consistently downregulated in BC tissues compared to normal tissues in almost all subtypes of BC (Li et al., 2011; Ouyang et al., 2014; Tahiri et al., 2014; Tashkandi et al., 2015; Cecene et al., 2016; Thakur et al., 2016; Adhami et al., 2018). The methylation state of CpG islands upstream of the miR-195/497 gene was found to be responsible for the downregulation of both miRNAs (Li et al., 2011). However, levels and activity of miR-195 were recently shown to be also dependent on a sponging circular RNA, circAGFG1, in triple-negative breast cancer; of note, circAGFG1 is able to cause Cyclin E1 (CCNE1) upregulation as a consequence of miR-195 sequestering (Yang et al., 2019). miR-503 has been also reported as downregulated in breast cancer and to confer sensibility to chemotherapy treatment (Gong et al., 2014; Long et al., 2015).

From a functional point of view, it has been reported that forced expression of miR-195 or miR-497 suppressed breast cancer cell proliferation and invasion. Raf-1 and CCND1 were identified as direct targets of miR-195 and miR-497 in BC cells (Li et al., 2011; Yang et al., 2013). Importantly, in the same study, miR-195/497 expression levels in clinical specimens were inversely correlated with malignancy of breast cancer (Li et al., 2011). Additional studies reported the ability of miR-195 and miR-497 to downregulate CCNE1 and CCND1 in BC cells (Hannafon et al., 2011; Luo et al., 2013, 2014). Interestingly, miR-497 has been also reported to target estrogen-related receptor alpha (ERRα), a nuclear receptor overexpressed in ERα negative breast cancer; downregulation of miR-497 is then responsible for ERRα induction and increased proliferation of triple-negative breast cancer cells (Han et al., 2016). Enforced miR-497 expression is also able to cause reduction of SMAD7, suppressing MDA-MB-231 and MCF-7 breast cancer cell growth (Liu et al., 2016). According to what was reported by Li et al. (2011); Liu et al. (2016) showed that high expression of miR-497 confers a better prognosis, indicated by the Kaplan–Meier test, especially in HER2 overexpressing and triple-negative breast cancer (TNBC).

With regard to additional functions, besides cell cycle control exerted in BC by miR-497, this miRNA has been shown to target HIF1A and SLUG, leading to the negative regulation of, respectively, angiogenesis and epithelial-to-mesenchymal transition (Wu et al., 2016a, b). miR-497 downregulation in BC was also shown to release BCL2 and BCL-W expression, thus inhibiting apoptosis (Shen et al., 2012; Wei et al., 2015). Expression of BCL2 in BC is also controlled by miR-195, whose overexpression enhances the sensibility of BC cells to both chemo- and radio-therapy (Singh and Saini, 2012; Zhu et al., 2015). miR-195 as well as miR-15a/miR-16 also impinges on the expression of fatty acid synthase (FASN), leading to the impairment in fatty acid synthesis pathway (Singh et al., 2015; Wang et al., 2016). Involvement of other members of the family, namely, miR-107 and miR-103, in the control of lipid metabolism had been previously shown by Wilfred et al. (2007), while experimental evidence demonstrating the ability of miR-107 to target FASN was also provided in normal and transformed hepatocytes (Bhatia et al., 2014).

Unexpectedly, and contrarily to miR-497, miR-195, and miR-503 that have been found consistently downregulated in BC, an increasing number of conflicting results is emerging with regard to the expression and function of other members of this group, for example, miR-103 and miR-107. These miRNAs, indeed, have been shown to exert oncogenic functions in the context of BC. Specifically, Martello et al. (2010) reported that miR-103 and miR-107 inhibit the expression of a key component of the miRNA processing machinery, Dicer, finally favoring the metastatic capacity of BC cells; this effect is obtained also as a result of the miR-200 family dowregulation caused by decreased Dicer activity. Coherently with this molecular network, authors show that high levels of miR-103/107 are associated with metastasis and poor outcome in BC. In agreement with the oncogenic model proposed by Martello et al., miR-103 and miR-107 were also shown to favor genomic instability through their ability to downregulate key players of DNA repair, as BRCA1 and RAD51 (Huang et al., 2013; Quann et al., 2015). An oncogenic network has been also evidenced, whereby miR-107 promotes tumor progression by targeting the tumor suppressor miRNA let-7 (Chen et al., 2011).

Despite these results, there is also literature highlighting the tumor suppressive function of miR-107 in BC. An interesting study from Polytarchou et al. (2012), for example, identifies miR-103/107 and miR-15/16 as microRNAs that are downregulated in cancer stem cells (CSCs) and that inhibit the growth of CSCs. These miRNAs were identified among other miRNAs previously reported as tumor suppressors. Authors show that miRNAs downregulated in CSCs affect common target genes that encode the Bmi1 and Suz12 components of the polycomb repressor complexes (PRCs) as well as the DNA-binding transcription factors Zeb1, Zeb2, and Klf4. Of note, they also show that an inverse relationship is present between the levels of CSC-regulating miRNAs and their respective targets in samples from triple-negative breast cancer patients, providing evidence for the relevance of these interactions in human cancer.

We found evidence in a recent study that the downregulation of miR-107 and of other members of the miR-15/107 group, namely, miR-15b and miR-195, in tumor-associated macrophages (TAMs), participate in the reprogramming of these cells in a pro-angiogenic sense. Specifically, miR-107, miR-15b, and miR-195 are decreased after culturing of macrophages with conditioned media from BC cells. This downregulation depends on the presence of ID4 protein in breast cancer cells as well as of VEGF in the conditioned medium. Functionally, the downregulation of these miRNAs enables released expression of proangiogenic factors, being Granulin (GRN) the most markedly affected (Donzelli et al., 2018). GRN had been previously demonstrated to be targeted by the miR-15/107 group in human cancer (Wang et al., 2010a, b). This soluble factor is attracting increasing interest due to its involvement in the regulation of tumor stroma function. GRN is associated with poor prognosis in BC (Elkabets et al., 2011; Yeh et al., 2015), and specifically in triple-negative breast cancer, a population of bone marrow cells secretes GRN to support stromal activation and robust tumor growth in young mice (Marsh et al., 2016). Interestingly, GRN has been recently reported as a key player also in pancreatic cancer metastasis, where macrophage-derived GRN induces liver fibrosis and contributes to cytotoxic CD8+ T-cell exclusion in metastatic livers (Nielsen et al., 2016; Quaranta et al., 2018).

The above described results suggest that members of miR-15/107 that exert an oncogenic function in BC cells, such as miR-107, might, on the contrary, act as tumor suppressors when expressed in cells of the tumor stroma, such as macrophages, or in the cancer stem cell compartment, highlighting the importance of the cell context for miRNA-associated functions.

### miR-15/107 as Biomarkers for Breast Cancer in Liquid Biopsy

Using circulating miRNAs as biomarkers has gained tremendous research interests. They are usually incorporated into exosomes or extracellular vesicles (EVs), secreted by cells, transferred to body fluids, and delivered into recipient cells, profoundly impacting the expression profile of these last. While extremely relevant for the understanding of cancer biology, these circulating miRNAs constitute, at the same time, a new category of potential non-invasive disease markers. With regard to miR-15/107 group, a growing body of evidence shows the potential of miR-195 as non-invasive biomarker for diagnosis and prognosis definition in breast cancer (Figure 1C). miR-195 has been found upregulated in the majority of studies analyzing liquid biopsies from BC patients. A meta-analysis by Liu et al. (2018), which analyzed six studies with a total of 464 patients and 287 healthy controls highlighted that miR-195 is suitable as a potential biomarker for early diagnosis of breast cancer with high sensitivity and specificity. A summary of the studies reported in literature is included in Table 1.

**TABLE 1:** Studies reporting the analysis of circulating miR-15/107 group members in breast cancer patients.

miRNA	Disease	Sample	Expression change (notes)	References
miR-195-5p	BC	Serum	up (vs. healthy donors)	Peña-Cano et al., 2019
miR-195-5p and miR-15a-5p	TNBC	Plasma	up (vs. healthy donors)	Qattan et al., 2017
miR-195-5p	BC	Serum	up (vs. healthy donors)	Fan et al., 2018
miR-195-5p	BC	Whole blood	up (vs. healthy donors and other cancers)	Heneghan et al., 2010
miR-195-5p	BC	Whole blood	up (high in postoperative patients with relapse)	Igglezou et al., 2014
miR-195-5p	BC	Plasma	down (vs. healthy donors)	Nadeem et al., 2017
miR-195-5p	BC	Serum	down (vs. healthy donors) up (after neoadj. chemo)	Zhao et al., 2014
miR-195-5p	TNBC	Serum	down (vs. triple positive BC)	Thakur et al., 2016
miR-103 and miR-107	TNBC	Serum	up (relapse vs. no relapse)	Kleivi Sahlberg et al., 2015
miR-424	BC	Serum	up (vs. healthy donors)	Zhang et al., 2015
miR-15a and miR-107	ER + BC	Serum	up (vs. healthy donors)	Kodahl et al., 2014

As already mentioned in the previous section, miR-195 expression is downregulated in BC tissues, compared to their normal tissue counterparts. On this basis, Qattan and colleagues hypothesized that cancerous cells may selectively export tumor suppressor miR-195 in order to maintain their oncogenic features (Qattan et al., 2017). This is an extremely interesting possibility that merits further investigation to be definitely proven. However, despite this, a couple of studies identified decreased miR-195 serum levels in BC patients compared to healthy controls (Zhao et al., 2014; Nadeem et al., 2017).

## Conclusion

microRNAs belonging to the miR-15/107 group are characterized by the presence of the “AGCAGC” hexamer in the “seed” region starting at either the first nucleotide or the second nucleotide from the 5′ end of the mature miRNA. This is an important determinant for target recognition and enables these miRNAs to recognize a huge number of common targets, mainly involved in the promotion of cell cycle progression, negatively regulating proliferation. Despite this, different members of this group may exert opposite functions in cancer cells, behaving as tumor suppressors or oncogenes, suggesting that the transformed context dictates their ability to recognize different panels of target mRNAs. The strong functional impact of the members of the miR-15/107 group on the proliferative potential of cancer cells makes them ideal candidates for the development of novel miRNA-based replacement strategies for the treatment of cancer. In addition, their presence in liquid biopsy samples from cancer patients prompts further investigations aimed at evaluating their usefulness for the monitoring of disease progression during follow-up.

## Author Contributions

All authors have contributed to the manuscript writing.

## Conflict of Interest

The authors declare that the research was conducted in the absence of any commercial or financial relationships that could be construed as a potential conflict of interest.
